# A genotypically distinct, melanic variant of *Anopheles arabiensi*s in Sudan is associated with arid environments

**DOI:** 10.1186/1475-2875-13-492

**Published:** 2014-12-13

**Authors:** Mariam Aboud, Abdelrafie Makhawi, Andrea Verardi, Fathi El Raba’a, Dia-Eldin Elnaiem, Harold Townson

**Affiliations:** Department of Biology, Faculty of Science and Technology, Al-Neelain University, Khartoum, Sudan; Department of Biotechnology, College of Applied and Industrial Sciences, University of Bahri, Khartoum, Sudan; Liverpool School of Tropical Medicine, Pembroke Place, Liverpool, L3 5QA UK; Department of Zoology, University of Khartoum, P.O. Box 321, Khartoum, Sudan; Department of Natural Sciences, University of Maryland Eastern Shore, 1 Backbone Rd, Princess Anne, MD 20851 USA

**Keywords:** *Anopheles arabiensis*, ND5, Sudan, Melanic, Malaria, Arid environments

## Abstract

**Background:**

*Anopheles arabiensis*, an important malaria vector in Sudan and other countries in sub-Saharan Africa, exhibits considerable ecological and behavioural plasticity allowing it to survive in the harsh conditions of arid regions. It has been shown that adult populations of *An. arabiensis* in the semi-desert habitat of western Khartoum State survive through the long dry season in a state of partial aestivation, characterized by limited feeding activity and a degree of arrested ovarian development. *Anopheles arabiensis* in these sites occurs in two phenotypic forms. One is large and heavily melanized, the other has the typical characteristics of *An. arabiensis* as found elsewhere in Africa. The extent of genetic variation in these forms was examined in widely separated locations in Sudan, including Kassala, Gedaref and the Northern States between 1998 and 1999 and 2004 and 2006.

**Methods:**

Each mosquito specimen was identified using standard morphological keys and a species-specific PCR test. Sequence variation in a 660 bp fragment of the mtDNA ND5 coding region was examined and the extent of genetic divergence between the forms was estimated from F_ST_ values using DNASP version 4.9. TCS 1.13 software was used to determine the genealogical relationships and to reflect clustering among mtDNA haplotypes.

**Results:**

The melanic and normal forms were found in sympatry in Kassala, Gedaref and Khartoum states, with the melanic form commonest in the hottest and most arid areas. Both forms were encountered in the periods of study: 1998–1999, and 2004–2006. Only ten specimens of *An. arabiensis* were collected from the Northern State in February 2006, all of which were of the normal form.

Based on the ND5 analysis, there was a marked subdivision between the normal and melanic forms (F_ST_ = 0.59). Furthermore, the melanic form showed more genetic variability, as measured by haplotype diversity (0.95) compared with the normal form (0.57), suggesting larger effective population.

**Conclusions:**

This is the first demonstration of correspondent phenotypic and genetic structuring in *An. arabiensis.* The high level of genetic differentiation shown by the mtDNA ND5 locus suggests that the two forms may represent separate species. It is hypothesized that the melanic form is better adapted to hot and arid environments.

**Electronic supplementary material:**

The online version of this article (doi:10.1186/1475-2875-13-492) contains supplementary material, which is available to authorized users.

## Background

The main groups of malaria vectors in Africa are *Anopheles gambiae*, *Anopheles funestus*, *Anopheles nili* and *Anopheles moucheti*. Each of these comprise a complex or group of genetically distinct species that are similar in morphology, but vary in traits that affect their role in transmission of malaria [1–3]. The most important of these groups is the *An. gambiae* complex, which comprises eight closely related species that are distributed through sub-Saharan Africa and its outer islands [4–7]. Within this complex, *An. gambiae*, *Anopheles coluzzii* and *Anopheles arabiensis* are the most efficient vectors of human malaria in Africa [5, 8, 9].

*Anopheles arabiensis* is a common malaria vector throughout sub-Saharan Africa [10–12]. This species shows considerable ecological and behavioural plasticity that allows it to survive in the harsh conditions of some arid areas. Some studies have indicated that *An. arabiensis* may be replacing *An. gambiae s.s.* as the dominant malaria vector in areas of East Africa, where insecticide-impregnated nets are used intensively [13, 14]. Different geographical populations of *An. arabiensis* show marked variations in their anthropophilic, exophilic and exophagic behaviour; thus adding more complexity to malaria transmission, and ultimately malaria control [15–19]. However, studies using a number of molecular markers, including partial mitochondrial gene sequences from the cytochrome b, ND1 and ND5 genes, microsatellite loci, chromosomal inversions and internal transcribed spacers 1 and 2 (ITS1 & ITS2) showed little population subdivision within *An. arabiensis* populations, as compared to *An. gambiae s.s.* [20–35]. This low level of population differentiation across the range of *An. arabiensis* populations has been attributed to recent population range expansion [28, 29].

In contrast, strong genetic differentiation in mtDNA (ND5) was detected between allopatric populations of *An. arabiensis* from the island of Reunion and the African continent, which was attributed to the low effective population size (Ne) on the island [32]. Lee *et al.* [36] reported a fixed nucleotide on the X chromosome between populations in East-southern Africa and those in Central Africa*.*

*Anopheles arabiensis* is the primary malaria vector throughout much of Sudan [37]. Other *Anopheles* species, such as *An. funestus, An. nili, Anopheles pharoensis, Anopheles rufipes* and *Anopheles dthali* are also present in the country, but they play a negligible role in malaria transmission. Malaria transmitted by *An. arabiensis*, continues to be a major health problem in Sudan [38, 39]. It has long been postulated that populations of *An. arabiensis* undergo aestivation during the dry season [40, 41]. It has been suggested that adults of a local population of *An. arabiensis*, in semi-desert habitat in Khartoum state (Sudan) are highly adapted to survive through the harsh long dry season, in a state of partial aestivation “with limited feeding activities and a degree of arrested ovarian development”. Similar findings were recently reported for *An. gambiae* and *An. coluzzii* (formerly S and M molecular forms) in Mali [42–46]. Interestingly, these studies showed that whereas aestivation is a dry season survival strategy used by the M form of *An. gambiae*, populations of *An. arabiensis* and the S form of *An. gambiae* from the same area are more likely to rely on migration from distant locations [43].

It is hypothesized that the persistence of *An. arabiensis* populations in the arid areas in Khartoum State may be reflected in the genetic structure of these populations. To test this hypothesis, a molecular study was conducted on adults of *An. arabiensis* collected from irrigated sites along the White Nile and from an arid region, West of Khartoum, where previous studies showed the presence of aestivating adults of this species [40]. In these regions adults of *An. arabiensis* occur in two phenotypic forms, distinguishable by their size and extent of melanization.

### Methods and study sites

**Figure 1:**
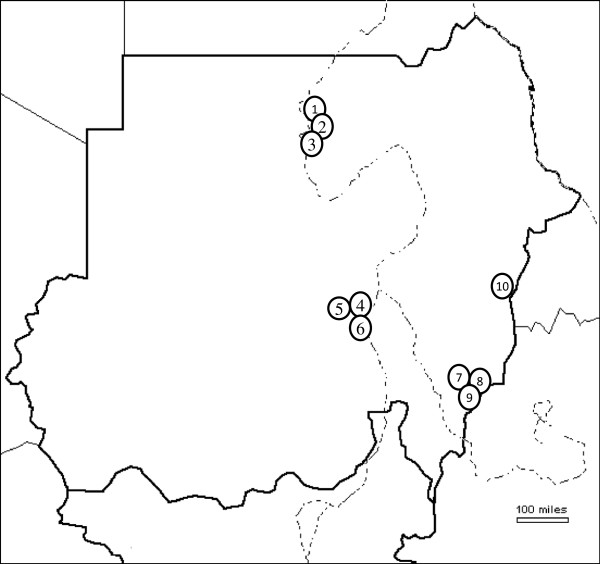
**Map of Sudan showing sampling sites collection of** ***Anopheles arabiensis*** **.** North State (sites 1,2 & 3); Khartoum (sites 4,5&6); Gedaref (sites 7,8&9) and Kassala (site 10).

Mosquito adult samples were collected from irrigated and non-irrigated habitats in Khartoum State (Central Sudan), Gedaref State (eastern Sudan), Kassala State (eastern Sudan) and Northern State. These locations are characterized by marked variations in rain precipitation and malaria endemicity (Figure 1 and Table 1). Whereas Gedaref and Kassala States are in the zone of hyper-endemic malaria, Khartoum and Northern states are considered to be hypo-, meso-endemic or zones free from malaria, respectively [39].

**Table 1:** **Sites, dates and sampling methods of adult mosquitoes used in study of genetic and morphometric differentiation of** ***Anopheles arabiensis*** **in Sudan 1998–2001 and 2004-2006**

Location	Position	Malaria endemicity	Rainy season	Rain precipitate	Sampling date	Sampling methods
Khartoum	15° 3'– 15° 45' N 32° 15' - 32° 45' E	hypo-meso endemic	July -September	240 mm	Nov 1998 - Oct 1999, July 2001, Dec 2004 – Nov 2006	Pyrethrum spray collection, Landing collection, Active search
Gedaref	12^°^ 54’N 35^°^ 08' E	hyper-endemic	June -October	815 mm	Dec 2004 and Nov 2006	Pyrethrum spray collection, Landing collection, Active search
Kassala	15° 22 N' 36° 27' E	hyper-endemic	June -October	400 mm	March 2005 and April 2006	Pyrethrum spray collection, Landing collection, Active search
North	19 ^°^ 16 N' 0° 27' E	free zones of malaria	September	1 mm	Feb 2006	Pyrethrum spray collection, Landing collection, Active search

#### Khartoum state

Located in central Sudan, at the confluence of the White Nile and the Blue Nile, (15°30' – 15°45' N and 32°15' - 32° 45' E), Khartoum State is characterized by a long dry and hot season between October – June followed by a short rainy season (July – September) with an average annual precipitation of 240 mm. Khartoum State has poor dry desert scrub vegetation, except along the riverbanks and in irrigated agricultural schemes. Sites used for collection of mosquitoes in Khartoum State were dry non-irrigated areas and irrigated areas close to the Nile, White Nile and the Blue Nile. Mosquito samples were collected between December 2004 and November 2006 in three villages in western Omdurman: El Mowailih (dry, 15° 34' N - 32° 23' E), El Rawakeeb (very dry 15°30' N - 32°15'E) and El Teris (irrigated 15°23' N - 32° 48' E).

#### Gedaref state

This State extends over large areas in the savannah region of Eastern Sudan. The climate is a tropical continental climate with two seasons; rainy season lasting from June-October and dry season between November to May with an average min-max temperature of 34-40°C. The average annual rainfall is 815 mm. Mosquito samples were collected during December 2004 and November 2006 in three villages in Rahad province: Batta (12°53' N - 35° 7' E), Kuka (13°06' N – 34°57' E) and Tabaldia (12°53' N -35°9' E). These villages lie along eastern bank of the El Rahad River, which is a seasonal watercourse that flows during and after the rainy season, June – December. Thereafter it fragments into small water pools during the dry hot season. Gedaref State is characterized with rich vegetation, especially during the rainy season. The main trees and bushes found in the study area are *Balanites aegyptiaca*, *Acacia seyal*, *Acacia mellifera*, *Acacia nilotica*, *Acacia senegal*, *Combretum spp*., *Azadirachta indica* and *Ziziphus spina-christi*. In the most of the places, the surface of the ground covered by *Sorghum spp.*, *Schoenfeldia ssp.*, *Cynodon spp.*, *Aristida spp.*, *Cinchrus spp.,* and *Brachiara spp.* grass, which flourishes during the rainy and post-rainy season and dies out during the dry season.

#### Kassala state

Kassala State lies about 611 km East of Khartoum, at the edge of the semi-desert region of eastern Sudan, Gedaref State from the South and close to the border with Eritrea. The climate of the area is characterized by a hot dry season, which extends from March-June followed by a short rainy season (June – October) with an average annual rainfall of 400 mm. The vegetation of Kassala is similar to that found in Gadaref State, but with a lower vegetation density. Mosquito samples were collected during March 2005 and April 2006 from Wad Sherifi village (15°22’ N - 36°27’ E). The only source of water is well-water storage tank.

#### Northern state

Located in the northwest corner of Sudan bordering Egypt and Libya, the Northern State is an extremely dry desert, intercepted by a narrow stretch of seasonally flooded and irrigated areas around the Nile. Rainfall rarely exceeds an average of 1 mm per annum, in most years and the vegetation ranges from true desert to semi-desert scrub. Daily maximum temperatures are generally high reaching 47°C beween May and August, with desert temperatures cool at night, and a minimum temperature of c.25-29°C.

### Morphological and molecular identification of mosquitoes

Mosquitoes were identified morphologically using standard taxonomic keys [47, 48].

Since the only *An. gambiae s.l*. species found in the study areas is *An. arabiensis*, these taxonomic keys provided a sufficient tool for species identification. In order to confirm the identification of the *An. gambiae* complex to species level, a molecular diagnostic test was performed using the rDNA species-diagnostic PCR protocol [49] protocol. DNA was extracted from individual mosquitoes using a previously described method [50].

### Amplification and sequencing of a mitochondrial DNA fragment

To study molecular variation of *An. arabiensis* populations, a 655 bp fragment of the mitochondrial nicotinamide adenine dinucleotide dehydrogenase gene subunit 5 (NADH-ND5) coding region sequences of mitochondrial DNA was PCR amplified as previously described [20]. The PCR mix comprised 50 μl reaction volume containing 1 μL of 1:200 DNA dilution, 50 pmol primers, DMP3A = 5’-AGG ATG AGA TGG CTT AGG TT-3’; 19CL = 5’- CTT CCA CCA ATT ACT GCT ATA ACA G-3’. The PCR product was purified using QIAquick PCR purification Kit (QIAGEN). The purified PCR product and cycle sequencing was performed with the ABI PRISM Dye Terminator Cycle Sequencing Kit (Applied Biosystems). DNA sequences were assembled, and analysis of nucleotide sequences was performed using an ABI PRISM 377 (Applied Biosystems) automated sequencer following the manufacturer’s protocols. All sequences were deposited in NCBI GenBank (accession numbers KJ950294-KJ950360).

### Mitochondrial ND5 DNA analysis

Prior to analysis, the 655 bp fragment of mtDNA (ND5) was confirmed by blasting against the corresponding *An. gambiae* sequence ([51]; GeneBank accession No. L20934). Subsequently, ND5 sequences from individual mosquitoes were aligned using the Clustal W programme and subjected to genetic variation analysis. The frequency of each haplotype, haplotype diversity (Hd), average number of pairwise nucleotide differences (π) and average number of nucleotides segregating per site (S), the population mutation rate (2N*M*) based on number of segregating sites (θ) were computed using the program DnaSP version 4.9 [52]. Sequences of ND5 were tested for neutral evolution, using Tajima’s D test [53] and Fu & Li’s F tests [54]. The extent of nucleotide differentiation between *An. arabiensis* populations was calculated by estimating F_ST_ values.

### Gene genealogy network

TCS 1.13[55] was used to determine the genealogical relationships and inspect clustering among mtND5 haplotypes. This method is based on statistical parsimony network [56], and was chosen because it accepts the existence of ancestral haplotypes, which are assumed to be the most frequent haplotypes, according to coalescence theory [57].

## Results

Although all specimens used in the study were morphologically and molecularly typed as *An. arabiensis*, two phenotypic forms of *An. arabiensis* were recognizable. One of these forms was larger, markedly darker and more metallic in colour than the other, which appeared as typical *An. arabiensis*. These forms are here labelled as the melanic (M) and normal (N) forms, respectively; all specimens were labelled accordingly.

Adult specimens of *An. arabiensis* were easily assigned to one of the two forms and no intermediate phenotypes were observed. The difference in body size was noticeable and measured. The body size of the adult melanic form was clearly larger than the normal form (mean body size and wing length =3.25 ± 0.22 mm and 2.68 ± 0.078 for the melanic form and 2.71 ± 0.19 mm and 2.37 ± 0.09 mm for the normal form, respectively). Both melanization and body size appeared to be inheritable characters. In colonization experiments, both forms produced corresponding adult offspring. The spatial distribution of normal and melanic forms in the different sites in Khartoum State is shown in Table 2. It is clear that the melanic form occurred more commonly in the hottest and most arid areas than in the irrigated areas (Tables 2 and 3, and Additional file 1).

**Table 2:** **Spatial and season distribution of melanic and normal forms of** ***An. arabiensis*** **collected form Khartoum State (Sudan) in 1998–1999 and 2004-2006**

Study area	Form	Dry season (Oct 1998-June 1999)	Rainy season (Jul-Sep 1998)	Dry season (Oct-2004-Jun-2005)	Rainy season (July-Sep 2005)
El Tries (Irrigated)	Normal	118 (92.9%)	241 (97.6%)	230 (94.3%)	67 (78.8%)
	Melanic	9 (7.1%)	6 (2.4%)	14 (5.7%)	18 (21.2%)
	Total	127	247	244	85
El Mowailih (Dry-irrigated)	Normal	49 (41.2)	312 (75.7%)	42 (16.1)	208 (66.5%)
	Melanic	70 (57.8)	100 (24.3%	219 (83.9)	105 (33.5%)
	Total	119	412	261	313
Elrawakeeb (Very dry)	Normal	-	-	-	2 (6.9%)
	Melanic	-	-	-	27 (93.1%)
	Total	-	-	-	29

**Table 3:** **Site and habitat description of melanic and normal forms of** ***An. arabiensis*** **populations collected in different localities of Sudan in 1999–2000 and 2004-2006**

Location	Habitat Type	Main water source	Breeding sites	Normal haplotype	Melanic haplotype
**Khartoum**					
El Mowailih	Dry	Well- pumped closed tanks	Water leaking from Well-water tanks	1(19),2,6,9,12	18(3),19,21,24,26(2)27(3),34(2),36(2),39(2),40,48,49,51,52(2),53(3), 54(2),57(3),60,61,65(2),66(3),67(4)
El Rawakeeb	Very Dry	Old wells and Well-pumped closed tanks	No water pools due to Sandy soil, and high rate of evaporation, Probably breeding in old wells	1(5),7(5),12(2)	29,31,47,53(4),56,60,67(3)
El Tries	Irrigated	White Nile	Flooded irrigated canals from White Nile	1(8),7,	20,28,30,38(2),50,52,53, 55,58,63,67(3)
Others	Irrigated	Nile, White Nile and Blue Nile	Flooded irrigated canals from Nile, White Nile and Blue Nile	1(3),8,12(2),15(2),17(2),	23,25,30,
**Gedaref**					
Bata	Semi dry	River, River pools,	Rain pools and river pools	1(4)	33,66,67(2)
Kuka	irrigated	River, River pools	Rain pools and river pools	1(3)	19,21,46, 67(2)
Tabaldia	Semi dry	River, River pools	Rain pools and river pools	1(6),3,7,11,12(2),16	18(3),20,21,22,32,44,45(2),61(2),67(3)
**Kassala**					
WadSharefi	Dry	Wells and Well-pumped tanks	Water leaking from ell-water tanks	1(15)3,4,5,7,8,13,14,16(2),	20,21,22,29,35,36,37(2)41,42,43,46,57(4),59,61,62,64,66(3),67(9)
**North**					
Ardwan	Irrigated	Nile River	Flooded irrigated canals rom Nile	1(2), 7	No melanic haplotype
ElSeleim	Irrigated	Nile River	Flooded irrigated canals from Nile	1(3),7(2)	No melanic haplotype
Sadiac	Irrigated	Nile River	Flooded irrigated canals from Nile	1,7	No melanic haplotype

### Haplotype diversity and genetic variation in the mtND5 of *Anopheles arabiensis*

Genetic variation in the populations of *An. arabiensis* was studied by examining variation in a 655 bp (position 6896–7550) fragment of the mtND5 gene of *An. gambiae* [51], for a total of 232 *An. arabiensis* samples spanning the four study areas. Reference sequences have been deposited in GeneBank (accession numbers KJ950294-KJ950360). All polymorphic sites were silent codon sites and the direct sequencing revealed no characteristics of heteroplasmy. No insertion and deletion differences were found within any mtDNA sequences, confirming that the sequenced segment of ND5 gene represented mtDNA rather than pseudo genes or nuclear–transposed copies.

The results of Tajima’s D test [51] and Fu & Li’s F tests [54] on ND5 gene sequences of all *An. arabiensis* individuals examined in this study are shown in Table 4. Significant departure from the neutral theory was noticeable in each population of the melanic and normal forms, except in Kassala area. The deviation was higher in the normal form, indicating higher selection pressure in the melanic form.

**Table 4:** **Haplotypes and nucleotide diversity of mtND5 within melanic and normal forms of** ***An. arabiensis*** **populations collected from different sites in Sudan during 1998–2001 and 2004-2006**

Location	Form	N	S	H	Hd	π _nd_	θ _s_	θ	Taji ma D	Fu &Li
Khartoum	Normal	53	8	9	0.573 (0.076)	0.00127 (0.00024)	0.00303	-1.3213	-1.6101	-1.6681
	Melanic	69	29	35	0.959 (0.028)	0.00514 (0.0003)	0.0099	-1.0686	-1.5263	-1.5681
	All	122	33	44	0.907 (0.021)	0.0057 (0.00024)	0.0099	-2.6336*	-1.3054	-2.5164*
Gadaref	Normal	19	6	6	0.538 (0.133)	0.0011 (0.00036)	0.0026	-2.205	-1.8697*	-2.4356
	Melanic	24	17	13	0.902	0.00547 (0.00056)	0.0069	-1.0686	-0.7632	-1.1399
					0.046					
	All	43	21	19	0.883 (0.037)	0.00554	0.0078	-1.8960	-0.9456	-1.863
						0.00041				
Kassala	Normal	25	9	10	0.647 (0.110)	0.00133 (0.00032)	0.0040	-3.184**	-2.2181**	-3.738**
	Melanic	32	24	18	0.907 (0.039)	0.00488 (0.0007)	0.0095	-2.277	-1.7072	-2.4653
	All	57	32	28	0.905 (0.028)	0.0062 (0.00035)	0.00113	-2.908*	-1.4739	-2.8434*
North	Normal	10	1	2	0.556 (0.075)	0.00085 (0.00011)	0.0054	0.8042	0.8042	1.0688
	Melanic	none	none	none	none	none	none	none	none	none
Overall	Normal	107	17	17	0.591 (0.054)	0.00126 (0.00017)	0.00524	-3.1767*	-2.1472*	-3.3358*
	Melanic	125	39	50	0.943 (0.014)	0.0052 (0.0003)	0.0122	-2.1426	-1.7545	-2.3809*
	All	232	50	67	0.897 (0.016)	0.0057 (0.00018)	0.00137	-3.1918*	-1.71937	-3.0457**

n, the number of sequences; S, the number of segregating sites; h, number of haplotypes; Hd, haplotypes diversity; π, the average number of pairwise nucleotide differences ((nucleotide diversity); θs the average number of nucleotides segregating per site, θ = Average number of mutation per sequence. Values in parentheses are SE. **P > 0.1; *0.10 > P > 0.05.

A total of 67 mtDNA haplotypes were found among 232 *An. arabiensis* individuals, 35 of which represented a single individual (see Additional file 1). The melanic form differed from the normal form by the presence of one or more of the substitutions A,C,T,G that consisted of four substitutions in positions 7240 (A substitute G), 7360 (C substitute T), 7486 (T substitute C) and 7627 (G substitute A). Of these four sites, the main substitution that differentiated all normal haplotypes from the melanic form was the first G/A substitution in position 7240. High levels of mtDNA ND5 haplotype diversity and nucleotide sequence divergence were encountered between allopatric and sympatric populations of the normal and melanic forms of *An. arabiensis* populations in Khartoum, Gedaref and Kassala States (Table 4).

It was clear that, in each location, the haplotype diversity within melanic populations of *An. arabiensis* was higher than within normal populations and almost equal to the overall haplotype diversity of the two forms. This result is consistent with a smaller effective population size of the normal form. In North State, where the analysis was restricted to a small number of the normal form (10 specimens), the haplotype and nucleotide diversity of *A. arabiensis* population were 0.56 (P ≤ 0.001) and 0.00085 (P ≤ 0.001), respectively.

The data provide clear evidence of corresponding morphological and genetic structuring in populations of *An. arabiensis* in sympatry. The average level of mtDNA ND5 sequence divergence within Sudanese *An. arabiensis* populations is 0.57% (±0.0002) (Table 4). This strong population subdivision within *An. arabiensis* populations was also supported by the F_ST_ value of 0.59 (P ≤ 0.0001) between normal and melanic forms. This provides strong evidence for limited gene exchange (*Nm* = 0.36) between melanic and normal forms of *An. arabiensis*, even though the forms are sympatric (Table 5).

**Table 5:** **Genetic differentiation (F**_**ST**_ **) and Gene flow (N** ***m*** **) between normal and melanic forms of sympatric and allopatric populations of** ***An. arabiensis*** **captured in different localities in Sudan in 1999–2000 and 2004-2006**

State	Sympatric populations	Allopatric populations			
		State	Normal V melanic	Normal V Normal	Melanic v Melanic
	Fst (N ***m***)		Fst (N ***m***)	Fst (N ***m***)	Fst (N ***m***)
Khartoum	0.58 (0.36)	Khartoum V Kassala	0.635 (0.29)	0.028(17.26)	0.0313 (15.62)
	(0.0000)*		(0.0000)*	(0.0655)^ns^	(0.1454)^ns^
Kassala	0.65 (0.27)	Khartoum V Gedaref	0.597 (0.34)	0.0027 (185.5)	0.01095 (45.2)
	(0.0006)*		(0.0000)*	(0.377)^ns^	(0.1921)^ns^
Gedaref	0.546 (0.42)	Kassala V Gedaref	0.55 (0.41)	0.0157 (32.4)	0.022 (22.1)
	(0.0008)*		(0.0008)*	(0.6383)^ns^	(0.3355)^ns^

ns, not significant; *P ≤ 0.001.

### Mitochondrial ND5 genealogy estimation

**Figure 2:**
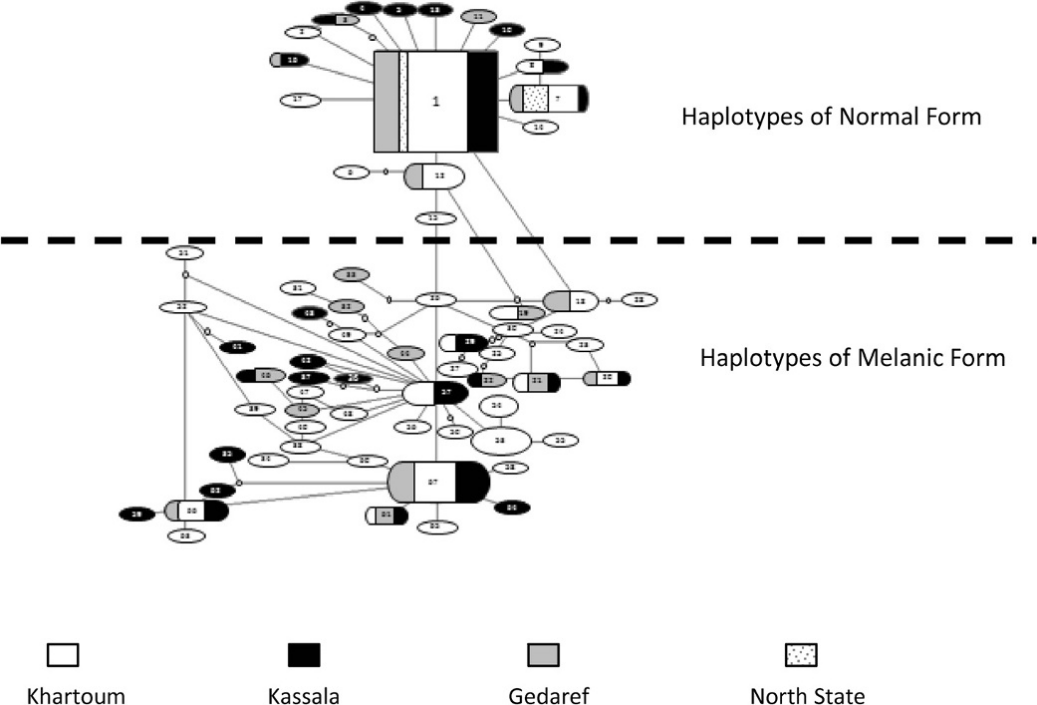
**Genealogical haplotype network analysis for a 655 bp fragment of ND5 of** ***An. arabiensis*** **in Sudan using the statistical parsimony method implemented in TCS (Clement et al.** [55]**).** Localities where haplotypes were found are represented by different fill patterns, as shown in the legend. Haplotype with the highest outgroup probability is displayed as rectangle and are assumed to be ancestral; other haplotypes are displayed as ovals. Haplotype symbol sizes correspond to frequencies; nodes without haplotype numbers correspond to assumed intermediates. Haplotypes number 1–17 represent a normal form of *An. arabiensis* and haplotypes number 18–67 represent melanic form of *An. arabiensis.*

In the Templeton network (Figure 2), the sequence data of mtND5 for *An. arabiensis* populations segregated into two major groups in addition to the minor varieties. Those two major groups of haplotypes correspond closely to the phenotypic variation detected within *An. arabiensis* populations, i.e. melanic and normal forms.

## Discussion

The findings obtained in this study provide first evidence of correspondent morphological and genetic population structuring in *An. arabiensis* that exist in sympatry. These results contradict the current notion that *An. arabiensis* populations in sub-Saharan Africa are panmictic [19, 20, 24, 26–31, 33, 34, 58]. Using relevant keys [47, 48] and species-diagnostic PCR method [49], all mosquitoes used in the study were morphologically and molecularly confirmed as *An. arabiensis*. However, two clearly distinguishable phenotypic forms were found: a typical normal colour-size form and a larger heavily melanized form. Furthermore, our observations lead us to hypothesize that the melanic form is more adapted to arid hot environment, as deduced from relative higher abundance and larger effective population size. This adaptation may have been conferred by the increased level of melanization, which is known to provide protection against desiccation [59–63].

From a molecular viewpoint, the normal form is identical to *An. arabiensis* type form based on colour and mtND5 sequence. The mtND5 gene of the melanic form is different from *An. arabiensis* type form by up to four nucleotide substitutions (one apparently fixed and three other variable nucleotide substitutions), although these forms are sympatric in different collection sites. These nucleotide substitutions, which were found in melanic form, are similar to those found in *An. arabiensis* populations in Senegal [20].

The average level of mtDNA ND5 sequence divergence within Sudanese *An. arabiensis* populations (0.57%) is higher than the average level of mtND5 sequence divergence within *An. gambiae* (0.38%) and *An. arabiensis* (0.46%) populations across Africa [20]. Moreover, the average level of mtDNA ND5 divergence between species (*An. gambiae* and *An. arabiensis*) was only 0.46% per nucleotide.

The F_ST_ value between sympatric populations of melanic and normal forms of *An. arabiensis* is significantly higher than previous F_ST_ pairwise studies of the same gene in *An. gambiae* and *An. arabiensis* populations in Africa, and other pairs of sibling species of Anopheles [20, 32, 36, 64]. Moreover, intraspecific estimates of genetic differentiation (F_ST_) within *An. arabiensis* populations were low (0.098) comparable to the interspecific F_ST_ estimates between *An. arabiensis* and *An. gambiae,* which were recorded as 0.07 and 0.12 for sympatric and allopatric populations, respectively [32].

The failure to find the melanic form in Northern State may be a consequence of the small sample size, or ecological separation due to the highly restrictive habitats of *An. arabiensis* in this area. Elsewhere in this study, the two forms were found in sympatry suggesting that there is a strong level of reproductive isolation between them. In this part of Sudan, where no rain may fall for several years, there is a sharp contrast between extreme desert conditions and irrigated areas along the Nile. The only suitable breeding and resting sites for *An. arabiensis* exist in agricultural schemes along the Nile or the ponds created by the seasonal flood in agricultural schemes. On the other hand away from the Nile there is no possibility of temporary mosquito breeding and resting sites due to lack of vegetation, extreme aridity and sandy soil topography of the area and in addition lack of well-pumped tanks. The few specimens of *An. arabiensis* collected in this study were captured during a short visit focusing on the irrigated area, where permanent breeding sites are present. Future studies should survey the area for possible presence of the habitat of the melanic form.

In dry areas of Khartoum and Kassala there is no surface water. The villagers obtain their water from a number of scattered water tanks which pumps water from capped wells. There are number of small scattered farms of sorghum around each water tank. *Anopheles arabiensis* populations breed in temporary breeding sites available from continuous leaking of water from these water tanks. The establishment of these well-pump tanks - which is the only available source of water for the villager’s uses - may serve as temporary breeding and resting sites for *An. arabiensis* in dry zone areas throughout the year. In Gedaref State, the study sites lie along eastern bank of El Rahad River, which is a seasonal watercourse that flows during and after the rainy season and then fragment into small water pools in the dry hot season that create temporary breeding sites.

The results of this study indicate that the melanic *An. arabiensis* are likely to be the mosquitoes said to have adapted to survive as adults through nine months of severe drought and heat [41]. This suggestion is based on the exclusive presence of this form during the hottest and driest parts of the year, its relatively larger size and its heavy melanization. Larger individuals should have smaller surface/volume ratio and, therefore, have higher resistance for desiccation. Larger individuals should also have higher accumulation of glycogen and lipids and, therefore, are more likely to increase their metabolic body water during dry conditions [59]. Furthermore, a number of recent studies demonstrated that melanization plays a major role in insect desiccation resistance by decreasing the permeability of the cuticle to water [59–62].

In *Drosophila melanogaster*, body melanization is a quantitative trait and shows significant levels of both within- and between-population variation [63]. Geographical populations of *D. melanogaster* from Africa, India and Australia exhibit clinal variation in melanization, which suggest adaptations to local climatic conditions [61]. Increased melanization has been associated with higher fitness under thermal as well as aridity stresses in *D. melanogaster*, *i.e.* a darker cuticle may improve thermoregulation as well as reduce cuticlar water loss [59, 61, 63]. The extreme dry zone areas appear to form barriers to gene flow between and among permanent wet irrigated and temporary breeding sites of dry areas *An. arabiensis* populations.

This study provides the first evidence of a high level of phenotypic and genetic sub-structuring of *An. arabiensis* in Sudan. This finding may have important implications for the ecology of *An. arabiensis* and the epidemiology and control of malaria in Sudan and other dry lands in Africa. In future studies, it will be important to examine other molecular markers and conduct mating studies to determine the degree of speciation between the two forms. Furthermore it would be interesting to compare the vector competence of the two forms and examine their role in malaria transmission. An understanding of the mechanisms and genes related to physiological adaptation in *An. arabiensis* may help explain how these mosquitoes survive through the dry season, and this in turn may help improve the control of malaria transmission in Sudan.

## Conclusions

This study provides the first evidence of a high level of phenotypic and genetic sub-structuring of *An. arabiensis* populations in Sudan. Whether this genetic differentiation is an indication of recent speciation process or the presence of two species is unclear. Judging from the higher level of genetic variability in the melanic mosquitoes, it may be inferred that they experience less selection pressure and, therefore are better adapted to dry hot conditions of the collection sites, a phenomenon that has recently been recognized in other insect species. The marked difference between the two forms may have significant consequences for malaria transmission and its control in the region.

## Electronic supplementary material

Additional file 1: **Polymorphic positions of mitochondrial DNA NAHD-dehydrogenase subunit 5 (ND5) gene in two forms of** ***An. arabiensis*** **collected from four collection sites (KS = Kassala, KH = Khartoum, GD = Gadaref, and NS = North State) with reference to the published mtDNA sequence (Beard** ***et al.*** [51]**; Gene Bank accession number L20934).** (DOC 37 KB)
