# Supplementary material for: A genotypically distinct, melanic variant of Anopheles arabiensis in Sudan is associated with arid environments
Source: Malar J. 2014 Dec 13;13:492. doi: 10.1186/1475-2875-13-492 (PMC4301653; doi:10.1186/1475-2875-13-492)
Supplement: Supplementary file 1 — Additional file 1: Polymorphic positions of mitochondrial DNA NAHD-dehydrogenase subunit 5 (ND5) gene in two forms of An. arabiensis collected from four collection sites (KS = Kassala, KH = Khartoum, GD = Gadaref, and NS = North State) with reference to the published mtDNA sequence (Beard et al. [51]; Gene Bank accession number L20934). (DOC 37 KB) [file 12936_2014_3672_MOESM1_ESM.doc]

Additional file 1 **Polymorphic positions of mitochondrial DNA NAHD-dehydrogenase subunit 5 (ND5) gene in two forms of *An. arabiensis* collected from four collection sites (KS=Kassala, KH=Khartoum, GD=Gadaref, and NS=North State) with reference to the published mtDNA sequence (Beard *et al*., 1993; Gene Bank accession number L20934).**

Polymorphic positions (ND5)

6 6 7 7 **7** 7 7 7 7 7 7 7 7 7 7 7 7 7 7 **7** 7 7 7 7 7 7 7 7 7 **7** 7 7 7 7 7 7 7 7 7 7 7 **7** 7 7 7 7 7 7 7 **7**

9 9 0 0 0 0 0 1 1 1 1 1 1 1 1 2 2 2 2 **2** 2 3 3 3 3 3 3 3 3 **3** 3 3 4 4 4 4 4 4 4 4 4 **4** 4 5 5 6 6 6 6 **6**

8 8 2 4 6 9 9 0 2 2 3 3 5 7 9 0 0 3 3 **4** 5 0 0 1 2 3 3 4 5 **6** 8 9 0 1 3 3 5 7 7 8 8 **8** 9 8 9 0 0 0 2 **2**

2 5 7 5 0 3 9 8 0 9 5 8 3 0 5 4 9 1 7 **0** 5 7 8 8 7 3 5 8 7 **0** 1 6 8 1 2 8 0 4 7 3 4 **6** 3 1 4 1 3 6 4 **7**

A A C **G** A A T A C A A T A C A T A C A **G** A G C A A A T T C **T** G A A A A A G T A C C **C** A A C C A G A **A**

**Localities**

KS KH GD NS n

**N***

15 34 13 05 . . . . . . . . . . . . . . . . . . . . . . . . . . . . . . . . . . . . . . . . . . . . . . . . . . 67

00 01 00 00 . . . . G . . . . . . . . . . . . . . . . . . . . . . . . . . . . . . . . . . . . . . . . . . . . . 01

01 00 01 00 . . . . . . . . T . . . . . . . G . . . . . . . . . . . . . . . . . . . . . . . . . . . . . . . . . 02

01 00 00 00 . . . . . . . . . G . . . . . . . . . . . . . . . . . . . . . . . . . . . . . . . . . . . . . . . . 01

01 00 00 00 . . . . . . . . . . G . . . . . . . . . . . . . . . . . . . . . . . . . . . . . . . . . . . . . . . 01

00 01 00 00 . . . . . . . . . . . . . . . A . . . . . . . . . . . . . . . . . . . G . . . . . T . . . . . . . . 01

01 06 01 05 . . . . . . . . . . . . . . . . . . . . T . . . . . . . . . . . . . . . . . . . . . . . . . . . . . 13

01 01 00 00 . . . . . . . . . . . . . . . . . . . . G . . . . . . . . . . . . . . . . . . . . . . . . . . . . . 02

00 01 00 00 . . . . . . . . . . . . . . . . . . . . G . . . . . . . . . . . . . . . . . . . A . . . . . . . . . 01

01 00 00 00 . . . . . . . . . . . . . . . . . . . . . A . . . . . . . . . . . . . . . . . . . . . . . . . . . . 01

00 00 01 00 . . . . . . . . . . . . . . . . . . . . . . . . G . . . . . . . . . . . . . . . . . . . . . . . . . 01

00 02 00 00 . . . . . . . . . . . . . . . . . . . . . . . . . . . . . C . . . . . . . . . . . T . . . . . . . . 02

01 00 00 00 . . . . . . . . . . . . . . . . . . . . . . . . . . . . . . . . . . G . . . . . . . . . . . . . . . 01

01 00 00 00 . . . . . . . . . . . . . . . . . . . . . . . . . . . . . . . . . . . . . C . . . . . . . . . . . . 01

00 05 02 00 . . . . . . . . . . . . . . . . . . . . . . . . . . . . . . . . . . . . . . . . . T . . . . . . . . 07

02 00 01 00 . . . . . . . . . . . . . . . . . . . . . . . . . . . . . . . . . . . . . . . . . . G . . . . . . . 03

00 02 00 00 . . . . . . . . . . . . . . . . . . . . . . . . . . . . . . . . . . . . . . . . . . . . . . G . . . 02

**M***

00 03 03 00 . . . . . . . . . . . . . . . . . . . A . . . . . . . . . . . . . . . . . . . . . . . . . . . . . . 06

00 02 00 00 . . . . . . . . . . . . . . . . . . . A . . . . . . . . . C . . . . . . . . . . . . . . . . . . . . 02

00 01 00 00 . . . . . . . . . . . . . . . . . . . A G . . G . . . . . C . . . . . . . . . . . . . . . . . . . . 01

01 01 01 00 . . . . . . . . . . . . . . . . . . . A G . . G . . . . . C . . . . T . . . . . . . . . . . . . . . 03

01 00 01 00 . . . . . . . . . . . . . . . . . . . A G . . . . . . . . C . . . . T . A . . . . . . . . . . . . . 02

01 01 02 00 . . . . . . . . . . . . . . . . . . . A G . . . . . . . . C . . . . T . . . . . . . . . . . . . . . 04

00 01 00 00 . . . . . . . . . . . . . . . . . . . A . . . . . . . C . C . . . . . . . . . . . . . . . . . . . . 01

00 01 00 00 . . . . . . . . . . . . . . . . . . . A . . . . . . . . . C . . . . . . A . . . . . . . . . . . . . 01

00 02 00 00 . . . . . . . . . . . . . . . . . . . A . . . . . . . . . C . . . . . . . . . . . T . . . . . . . . 02

00 03 00 00 T . . . . T . . . . . . . . . . . . . A . . . . . . . . . C A . . . . . . . . . . T . G . . . . . . 03

00 01 00 00 . . T . . . . . . . G . . . . . . . . A . . . . . . . . . . . . . . . . . . . . . . . . . . . . . . 01

01 01 00 00 . . . . . T . . . . . . . . . . . . . A . . . . . . . . . C A . . . . . . . . . . . . G . . . . . . 02

00 01 01 00 . . . . . . . . . . . C . . . . . . . A . . . . . . . . . . . . . . . . . . . . . T . . . . . . . . 02

00 01 00 00 . . . . . . A . . . . . . . . . . . . A . . . . . . . . . C A . . . . . A . . T . T . . . . . . . . 01

00 00 01 00 . . . . . . A . . . . . . . . . . . . A . . . . . . . . . C A . . . . . . . . T . T . . . . . . . . 01

00 00 01 00 . . . . . . . . T . . . . . . . . . . A . . . . . . A . . C . . . . . . . . . . . T . . . . . . . . 01

00 02 00 00 . . . A G . . . . . . . . . . . . . . A . . . . . . . . . C . . . . . . . . . . . T . . . . . . . . 02

01 00 00 00 . . . A . . . . . . . . . . . . . . . A . . . . . T . . . C . . . . . . . . . . . . . . . . . . . G 01

01 00 00 00 . . T . . . . . . . . . . . . . . T . A . . . . . . . . . C . . . . . . . . . . . T . . . . . . . G 01

02 00 00 00 . . T . . . C G . . . . . . . . . . . A . . . . . . . . . C . . . . . . . . . . . T . . . . . . . G 02

00 02 00 00 . . . . G . . . . . . . . . . . . . . A . . . . . . . . . C . . . . . . . . . . . T . . . . . . . G 02

00 02 00 00 . . . . G . . . . . . . . . . . . . . A G . . . . . . . . C . . . . . . . . . . . T . . . . . . . G 02

00 01 00 00 . . . . G . . . . . . . . . . . . . . A . . . . . . . . . C . G . . . . . . . . . T . . . . . . . G 01

01 00 00 00 . . . . . . . . T . . . . T . . . . . A G . . . . . . . . C . . . . . . . . . . . T . . . . . . . G 01

01 00 00 00 . . . . . . . . . . . . . . . . . T . A . . . . . . . . . C . . . . . . . . . . . T . . . . . . . G 01

01 00 00 00 . . . . . . . . . . . . . . . . . . G A . . . . . . . . . C . . . . G . . . . T . T . . . . . . . G 01

00 00 01 00 . . . . . . . . . . . . . . . . . . . A . . . . . . . . . C A . . . . . . . . . . T . . . . . . . G 01

00 00 02 00 . . . . . . . . . . . . . . . . . . . A . . . . . . . . . C . G . . . . . . . . . T . . . . . . . G 02

01 00 01 00 . . . . . . . . . . . . . . . . . . . A . . . . . . . . . C . G . . . . . . G . . T . . . . . . . G 02

00 01 00 00 . . . . . . . . . . . . . . . . . . . A . . . . . . . . . C . G . . . . A . . . . T . . . . . . . G 01

00 01 00 00 . . . . . . . . . . . . . . . . . . . A . . . . . . . . . C . . . . . . A . . . . T . . . . . . . G 01

00 01 00 00 . . . . . . . . . . . . . . . . . . . A . . . . . . . . . C . . . . . . . . . T . T . . . . . . . G 01

00 01 00 00 . . . . . . . . . . . . . . . . . . . A . . . . . . . . . C . . . . . . . . . . . T . . . . G . G G 01

00 01 00 00 . . . . . . . . . . . . . . . . . . . A T . . . . . . . . C . . . . . . . . . . . T . . T . . . . G 01

00 03 00 00 . . . . . . . . . . . . . . . . . . . A G . . . . . . . . C . . . . . . . . . . . T . . . . . . . G 03

00 08 00 00 . . . . . . . . . . . . . . . . . . . A . . . G . . . . . C . . . . . . . . . . . T . . . . . . . G 08

00 02 00 00 . . . . . . . . . . . . . . . . . . . A . . . G . . . . . C . . G . . . . . . . . T . . . . . . . G 02

00 01 00 00 . . . . . . . . . . . . . . . . . . . A . . . G . . . . . C . . . G . . . . . . . T . . . . . . . G 01

00 01 00 00 . . . . . . . . . . . . . . . . . . . A . . . . . . . . T C . . . . . . . . . . . T . . . . . . . G 01

04 03 00 00 . . . . . . . . . . . . . . . . . . . A . . . . . . . . . C . . . . . . . . . . . T . . . . . . . G 07

00 01 00 00 C . . A . . . . . . . . . . . . . . . A . . . . . . . . . C . . . . . . . . . . . T . . . . . . . G 01

01 00 00 00 . C . A . . . . . . . . . . . . . . . A G . . . . . . . . C . . . . . . . . . . . T . . . . . . . G 01

00 02 00 00 . . . A G . . . . . . . . . . . . . . A . . . . . . . . . C . . . . . . . . . . . T . . . . . . . G 02

01 01 02 00 . . . A . . . . . . . . . . C . . . . A . . . . . . . . . C . . . . . . . . . . . T . . . . . . . G 04

01 00 00 00 . . . A . . . . . . . . . . . . . . . A G . . . . T . . . C . . . . . . . . . . . T . . . . . . . G 01

00 01 00 00 . . . A . . . . . . . . . . . . . . . A G . . . . . . . . C . . . . . . . . . . . T . . . G . . . G 01

01 00 00 00 . . . A . . . . . . . . . . . . . . . A . . A . . . . . . C . . . . . . . . . . . T . . . . . . . G 01

00 02 00 00 . . . A . . . . . . . . . . . . . . . A . . . . . . . . . C . . . . . . . . . . . T . . . . . T . G 02

03 03 01 00 . . . A . . . . . . . . . . . . . . . A G . . . . . . . . C . . . . . . . . . . . T . . . . . . . G 07

09 10 07 00 . . . A . . . . . . . . . . . . . . . A . . . . . . . . . C . . . . . . . . . . . T . . . . . . . G 26

**Total 67/122/43/10 232**

N= Normal, M= Melanic, n= sample size. Dots represent identity with respect to the first sequence listed. The frequency (n) indicates the number of times the haplotype was found in each sample. Number of haplotypes in serial format from 1-67.
